# *In vitro* screening of 65 mycotoxins for insecticidal potential

**DOI:** 10.1371/journal.pone.0248772

**Published:** 2021-03-18

**Authors:** Mieczysława Irena Boguś, Anna Katarzyna Wrońska, Agata Kaczmarek, Martyna Boguś-Sobocińska

**Affiliations:** 1 Witold Stefański Institute of Parasitology, Polish Academy of Sciences, Warszawa, Poland; 2 Biomibo ul, Warszawa, Poland; Ghent University, BELGIUM

## Abstract

The economic losses and threats to human and animal health caused by insects and the pathogens transmitted by them require effective and environmentally-friendly methods of controlling them. One such group of natural biocontrol agents which may be used as biopesticides is that of the entomopathogenic fungi and their toxic secondary metabolites (mycotoxins). The present *in vitro* work examined the insecticidal potential of 65 commercially-available mycotoxins against the insect Sf-9 cell line. Mammalian Caco-2 and THP-1 cell lines served as reference controls to select insecticidal mycotoxins harmless to mammalian cells. All tested mycotoxins significantly reduced the *in vitro* proliferation of the Sf-9 cells and evoked morphological changes. Ten of the mycotoxins found to strongly inhibit Sf-9 proliferation also had moderate or no effect on Caco-2 cells. The THP-1 cells were highly resistant to the tested mycotoxins: doses 10^3^ times higher were needed to affect viability and morphology (1 μg/ml for THP-1 versus 1 ng/ml for Sf-9 and Caco-2). Nine mycotoxins significantly decreased Sf-9 cell proliferation with minor effects on mammalian cells: cyclosporins B and D, cytochalasin E, gliotoxin, HC toxin, paxilline, penitrem A, stachybotrylactam and verruculogen. These may be good candidates for future biopesticide formulations.

## Introduction

Mycotoxins constitute a group of varied compounds produced naturally by fungi as secondary metabolites which pose a risk to human and animal health and can cause a variety of ill effects from allergic responses to immunosuppression and cancer. They are typically not essential to the growth and reproduction of the producing organism [1]. Many mycotoxins suppress the immune functions of mammals by decreasing the proliferation of activated lymphocytes, impairing the phagocytic function of macrophages, modulating apoptosis, and suppressing cytokine production. Their impairment of immune-related organs alters the susceptibility of the host to the pathogens [1, 2]. In turn, many secondary metabolites produced by fungi play a role as pathogenicity or virulence factors in plants [3]. Fortunately, of over the 300 mycotoxins which have been identified, only a few regularly contaminate food and animal feed, and pose any serious risk to human and animal health. Hence, a number of studies are available on the occurrence and detection of the toxic effects of aflatoxins, ochratoxins, fumonisins, patulin and zearalenone, as well as trichothecenes such as deoxynivalenol and T2 toxin, and their mode of action against humans and animals [4–6]. Data on other identified mycotoxins appear sporadically.

Entomopathogenic fungi are natural enemies of insects, and their role in the regulation of insect populations is relatively well described [7]. In response to the need to reduce the amount of chemical insecticides, interest has been growing in the use of entomopathogenic fungi as bio-insecticides [8]. This interest stems from the fact that these organisms are naturally present in the environment, typically have a narrow host range, and as the mycotoxins produced in insect hosts have limited ways to enter the environment, there is little chance that they may contaminate foodstuffs [7–9]. Few mycotoxins produced by entomopathogenic fungi are currently commercially available: beauvericin produced by *Beauveria bassiana*, cytochalasin C produced by *Metarhizium anisopliae*, and cyclosporin H produced by *Tolypocladium inflatum*. A single mold species may produce many different mycotoxins, and several species may produce the same mycotoxin [3–5]; in addition, there are reports on the insecticidal properties of several mycotoxins [10–12] for which there is currently no information about their relationship with entomopathogenic fungi. Therefore, the present study examines whether commercially-available mycotoxins have the potential to be used as insecticides to control populations of harmful insects. It should be noted that several mycotoxins have already found applications in clinical medicine as antibiotics, growth promoters, and various drugs [13]. Most mycotoxins are very stable [14, 15]; however, while this may be an advantage when used in field conditions to combat insect pests, this may be associated with a risk of excessive accumulation in the environment. Fortunately, significant progress have been made in identifying microorganisms and microbial enzymes that can effectively degrade mycotoxins in an environmentally-friendly manner [16, 17], which offers hope for solving the problem of such accumulation. Although *in vivo* studies using insects provide useful information on toxicity toward the target organism, they are time consuming and demand the use of high levels of tested compounds. In contrast, *in vitro* cytotoxicity tests are less expensive, more reproducible, and much faster. The aim of this study was to identify commercially-available mycotoxins that could act as promising candidates for further studies on potential insecticides. A testable hypothesis was to check whether the commercially-available mycotoxins could affect the morphology and *in vitro* proliferation of insect cells. The study evaluates the sensitivity of the Sf-9 cell line from fall armyworm, *Spodoptera frugiperda*, an important crop pest, toward 65 mycotoxins and compares the findings with those obtained from two human cell lines, Caco-2 and THP-1, which are commonly used as *in vitro* mammalian models.

## Materials and methods

### Mycotoxins

The following mycotoxins on were administered to the Sf-9, Caco-2, and THP-1 cells cultures: 3-acetyldeoxynivalenol, aflatoxicol, aflatoxin B1, aflatoxin B2, aflatoxin G1, aflatoxin G2, aflatoxin M1, aflatoxin M2, alternariol, alternariol-9-methyl ether, α-amanitin, β-amanitin, γ-amanitin, antibiotic PF 1052, apicidin, beauvericin, brefeldin A, chaetocin, citreoviridin, citrinin, cyclopiazonic acid, cyclosporin A, cyclosporin B, cyclosporin C, cyclosporin D, cyclosporin H, cytochalasin A, cytochalasin B, cytochalasin C, cytochalasin D, cytochalasin E, deoxynivalenol, diacetoxyscirpenol, fumagillin, fumigaclavine A, fumonisin B1, fumonisin B2, fusarenon X, gliotoxin, HC toxin, HT-2-toxin, moniliformin, moniliformin sodium salt, mycophenolic acid, neosolaniol, ochratoxin A, ochratoxin B, patulin, paxilline, penitrem A, phomopsin A, roquefortine C, skyrin, stachybotrylactam, sterigmatocystin, strobilurin B, T2 tetraol, T2 toxin, T2 triol, tenuazonic acid, territrem B, verruculogen, wortmannin, zearalenone, and α-zearalanol. Mycotoxins were purchased via Axxora platform (http://www.axxora.com). All mycotoxins were dissolved in 99.8% ethanol (POCH) for use in *in vitro* tests.

### Culture of Sf-9 cells

The Sf-9 cell line from *Spodoptera frugiperda* pupal ovarian tissue (Thermo Fisher Scientific) was cultured in Gibco Grace’s Insect Medium (Thermo Fisher Scientific) supplemented with 10% Fetal Bovine Serum (FBS; Thermo Fisher Scientific), 10 mg/ml gentamycin (Sigma Aldrich) and 250 μg/ml amphotericin B (Sigma Aldrich). To examine the effect of the mycotoxins, the cells were transferred to 24-well plates (Nest). The cell density was 1 x 10^6^ cells/well in 400 μl. After 24-hour incubation at 27°C, the mycotoxins dissolved in ethanol were added to the cells. The final concentration of toxin in the well was 1 ng/ml. The dose was selected on the basis of pilot studies in which various concentrations (0.1–50 ng/ml) were tested on SF-9 cells (not shown). The cells were incubated for 24 hours in optimal conditions. After that time, the effect of the toxins on the cells was examined. Two negative controls were used: C1- untreated cells and C2—cells that received only ethanol (1% per well). Each control was performed in 20 independent replications. The effect of each mycotoxin on Sf-9 cell line was tested in 3 independent replications performed separately for proliferation assays and for microscopy.

### Culture of Caco-2 cells

A Caco-2 continuous line of heterogeneous human epithelial colorectal adenocarcinoma cells were purchased in Sigma Aldrich. The cells were cultured in Dulbecco modified Eagle medium (DMEM; Merck) supplemented with 10% Fetal Bovine Serum (FBS; Merck), 10 mg/ml gentamycin (Sigma Aldrich) and 250 μg/ml amphotericin B (Sigma Aldrich). The cells were grown in 25 cm^2^ culture flasks (Nest) and incubated at 37°C in a humidified atmosphere with 5% CO_2_. The cell line was subcultured at 70–80% confluence. Briefly, the cells were detached with 0.25% trypsin / 0.02% sodium EDTA solution (Merck) and split 1: 3–1: 6 (i.e., seeded at a density of 2–4 × 10^5^ cells/cm^2^).

To examine the effects of the mycotoxins, the cells were seeded in 24-well plates (Nest) at a density of 1.2 × 10^5^ cells/well and allowed to grow in 400 μl medium until confluence. Mycotoxins dissolved in ethanol were added to the cells at a final concentration of 1 ng/ml. The dose was selected on the basis of pilot studies in which various concentrations (0.1–50 ng/ml) were tested on Caco-2 cells (not shown). The cells were incubated for 24 hours under optimal conditions (37°C in a humidified atmosphere with 5% CO_2_). After that time, the effect of toxins on cells was examined. Two negative controls were used: C1- untreated cells, and C2—cells that received only ethanol (1% per well). Each control was performed in 20 independent replications. The effect of each mycotoxin on Caco-2 cell line was tested in 3 independent replications performed separately for proliferation assays and for microscopy.

### Culture of THP-1 cells

THP-1 cells (Sigma Aldrich), a pro-monocytic cell line, were cultured in RPMI 1640 (Merck) supplemented with 10% Fetal Bovine Serum (FBS; Merck), 10 mg/ml gentamycin (Sigma Aldrich) and 250 μg/ml amphotericin B (Sigma Aldrich). The cells were seeded at a density of 7 x 10^5^ per well in a 24-well plate (Nest), to a total of 400 μl medium and incubated at 37°C in a humidified atmosphere of 5% CO_2_ for 24 hours. After this time, the test mycotoxins diluted in ethanol were added to the cells.

**Fig 1:**
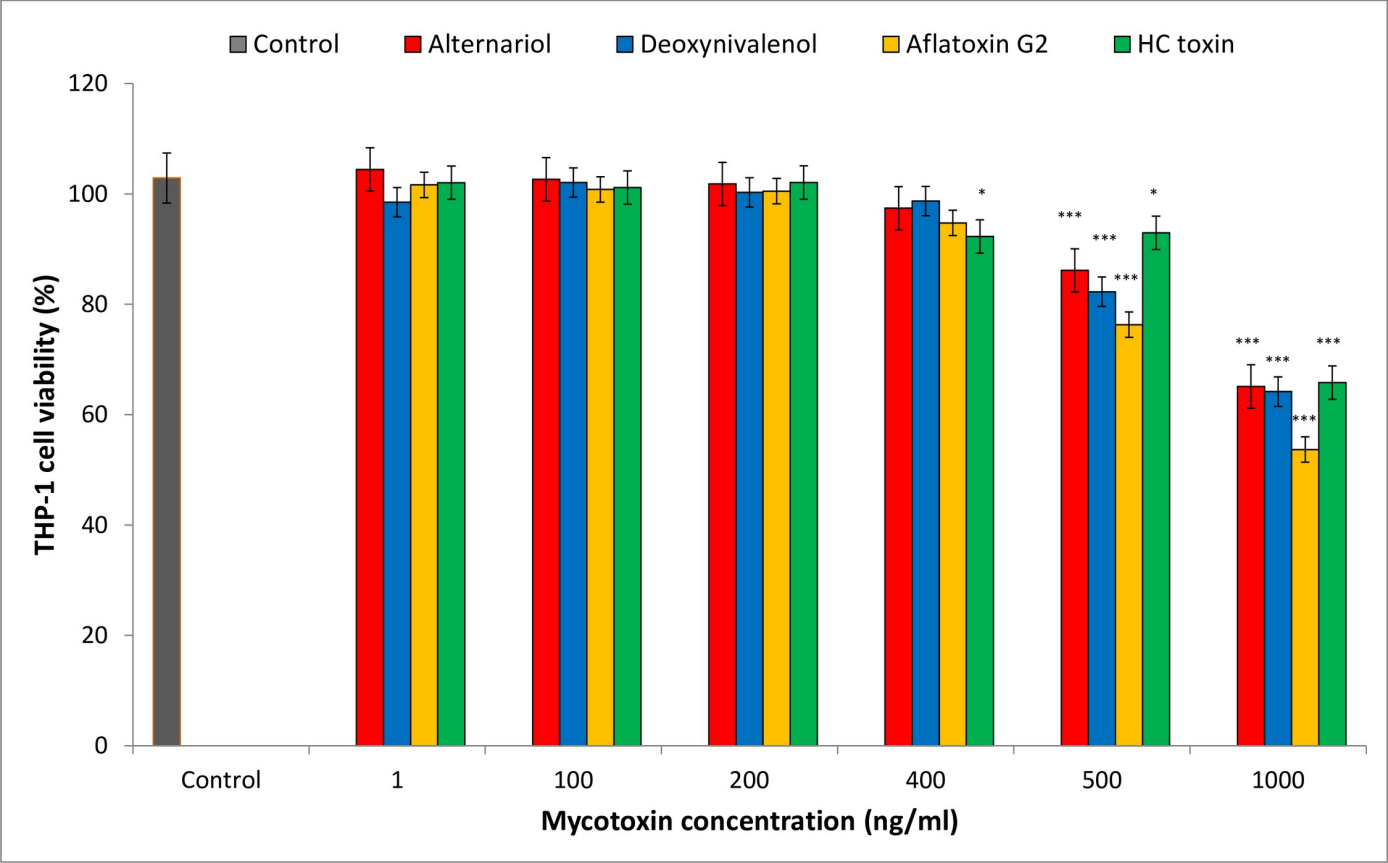
Dose-response effects of four mycotoxins on the viability of THP-1 cells *in vitro*. The statistical significance of the differences observed between treated cultures and controls at all concentrations of each tested mycotoxin were tested using Dunnett test. Each test was performed in 3 independent replications; control C2 (solvent treatment) in 20 replications. Statistical significance: * p < 0.05, ** p <0.001, *** p <0.0001.

As the THP-1 cells displayed high resistance to mycotoxins administered at a concentration of 1 ng/ml during preliminary studies, four randomly-selected mycotoxins (alternariol, deoxynivalenol, aflatoxin G2 and HC toxin) were added at various concentrations to determine appropriate test concentrations. The mycotoxins, dissolved in ethanol, were added to the cells to give the following final concentrations per well: 1 ng/ml, 100 ng/ml, 200 ng/ml, 400 ng/ml, 500 ng/ml and 1 μg/ml. The results of this trial (Fig 1) indicated that 1 μg/ml would be an appropriate concentration for testing cell proliferation and viability. After addition of 1μg/ml mycotoxins, the THP-1 cells were incubated for 24 hours under optimal conditions (37°C in a humidified atmosphere of 5% CO_2_). Following this, the effect of the toxins on the cells was examined. Two negative controls were used: C1- untreated cells, and C2—cells that received only ethanol (1% per well). Each control was performed in 20 independent replications. The effect of each mycotoxin on THP-1 cell line was tested in 3 independent replications performed separately for proliferation and viability assays as well as for microscopy.

### Study of the mycotoxin effects on insect and mammalian cells

After 24h incubation of Sf-9, Caco-2, and THP-1 cells with tested mycotoxins, the ability of cells to proliferate were investigated and morphological changes were analyzed. In the THP-1 cultures, cell viability was additionally tested.

Cell Counting Kit-8 (CCK-8, Abbkine) was used to measure cell proliferation. This reagent based on WST-8 (water-soluble tetrazolium salt) is reduced by the dehydrogenases in cells to give an orange colored product (formazan). The amount of the formazan in cells was directly proportional to the number of living cells. Briefly, 40 μl of CCK-8 reagent was added to each well containing cell cultures (test or control), the plates were then incubated for four hours under the described above optimal conditions for each cell type. Absorbance readings were taken at 450 nm using a Synergy HT Multi-Mode Microplate Reader (BioTek). The results are presented as a percentage of control (C2) findings, assumed to be 100%.

Of the tested cells, only the THP-1 line is non-adherent. Therefore, it was possible to check the viability of these cells with propidium iodide (PI; Invitrogen). The final concentration of the reagent in the wells of culture plates was 10 μg/ml. After a 10-minute incubation period at room temperature, an Arthur cell counter (NanoEnTek) was used to calculate the number of cells. The results are presented as a percentage of dead cells. Similar attempts to detach adherent Sf-9 and Caco-2 cells, both mechanically and enzymatically, resulted in very high mortality, which prevented the measurement of cell viability in these cultures.

Phase contrast microscope images of control and treated Sf-9, Caco-2, and THP-1 cells were taken at various time points (from five minutes up to 24 hours after treatment) using a Axio Vert.A1 fluorescence inverted microscope (Zeiss) equipped with Axio Cam ICc 5 camera (Zeiss) and Zen lite 2012 software (Zeiss).

### Statistical analysis

Statistical analysis was performed using STATISTICA 6.1 software (StatSoft Polska). Any statistical relationships were evaluated using the one-way ANOVA, *t* test and Dunnett test for *post hoc* analysis. The Kolmogorov-Smirnov (K-S) test, skewness and kurtosis were used to check normality of controls. All data are shown as means ± SD, and values of *p* ≤ 0.05 were considered as significant.

## Results

### Effects of 65 tested mycotoxins on the proliferation and viability of insect and mammalian cells

From the data presented in Table 1, it appears that all tested mycotoxins significantly reduced *in vitro* proliferation of Sf-9 cells as compared with control cells (C2) which received an appropriate amount of solvent (ethanol). Regarding the Sf-9 cells the strongest effect (a fall in cell proliferation to 13–30% of that observed in control C2) was observed in the case of 24-hour incubation with 1 ng/ml of 19 mycotoxins: 3-acetyldeoxynivalenol, aflatoxins B1 and B2, alternariol, alternariol-9-methyl ether, beauvericin, brefeldin A, cyclosporin D, cytochalasin E, fumonisin B2, gliotoxin, ochratoxin A, patulin, paxilline, penitrem A, stachybotrylactam, sterigmatocystin, T2 tetraol, and verruculogen. In contrast, the weakest effect (cell proliferation drop to 70–91% of that observed in control C2) was detected in the case of 13 mycotoxins: aflatoxicol, aflatoxin M1, γ-amanitin, antibiotic PF 1052, citrinin, cytochalasin D, fumagillin, HT-2-toxin, roquefortine C, T2 triol, wortmannin, zearalenone, and α-zearalanol.

**Table 1:** *In vitro* effects of 65 mycotoxins on proliferation and mortality of Sf-9, Caco-2 and THP-1 cell lines.

Mycotoxin	CAS / Cat no.	Mycotoxin source	Proliferation of mycotoxin treated cells				Cell mortality
			Treated Sf-9 cells (1ng/ml) vs. control (% ± SD)	Statistical significance Sf-9 vs Caco-2	Treated Caco-2 cells (1ng/ml) vs. control (% ± SD)	Treated THP-1 cells (1μg/ml) vs. control (% ± SD)	Treated THP-1 cells (1μg/ml) vs. control (% ± SD)
Control C2			100 ± 6		102 ± 6	103 ± 7	10 ± 3
3-Acetyldeoxynivalenol	50722-38-8 / LKT-D1760-M001	*Fusarium graminearum*	24 ± 3 ***	**	64 ± 5 ***	138 ± 8 ***	6 ± 1
Aflatoxicol	29611-03-8 / ENZ-CHM104-0001	*Aspergillus flavus*	76 ± 3 ***	*	41 ± 5 ***	55 ± 3 ***	35 ± 3 ***
Aflatoxin B1	1162-65-8 / ALX-630-093-M001	*Aspergillus flavus*	25 ± 1 ***	**	48 ± 2 ***	65 ± 4 ***	25 ± 2 ***
Aflatoxin B2	7220-81-7 / ALX-630-103-M001	*Aspergillus flavus*	28 ± 1 ***	**	82 ± 5 **	103 ± 2	7 ± 1
Aflatoxin G1	1165-39-5 / ALX-630-104-M001	*Aspergillus flavus*	37 ± 2 ***	*	64 ± 5 ***	74 ± 2 ***	16 ± 2 *
Aflatoxin G2	7241-98-7 / ALX-630-106-M001	*Aspergillus flavus*	55 ± 2 ***	*	48 ± 2 ***	54 ± 2 ***	35 ± 3 ***
Aflatoxin M1	6795-23-9 / ALX-630-095-MC01	*Aspergillus flavus*	77 ± 1 ***		76 ± 6 ***	87 ± 2 *	25 ± 3 ***
Aflatoxin M2	6885-57-0 / ALX-630-114-MC01	*Aspergillus flavus*	55 ± 4 ***	**	104 ± 6	97 ± 2	12 ± 1
Alternariol	641-38-3 / ALX-350-139-M001	*Alternaria* sp.	15 ± 1 ***	***	97 ± 4	65 ± 4 ***	21 ± 1 ***
Alternariol-9-methyl ether	23452-05-3 / LKT-A4678-M001	*Alternaria* sp.	20 ± 1 ***	**	59 ± 4 ***	84 ± 2 **	18 ± 1 ***
α-Amanitin	23109-05-9 / ALX-350-270-M001	*Amanita phalloides*	56 ± 2 ***	*	47 ± 4 ***	93 ± 2 *	14 ± 1
β-Amanitin	21150-22-1 / ALX-350-271-M001	*Amanita phalloides*	46 ± 3 ***	*	61 ± 2 ***	84 ± 1 **	19 ± 1 ***
γ-Amanitin	21150-23-2 / ALX-350-272-M001	*Amanita phalloides*	86 ± 1 ***		87 ± 4	76 ± 2 ***	25 ± 2 ***
Antibiotic PF 1052	147317-15-5 / ALX-380-147-M001	*Phoma* sp.	74 ± 2 ***	*	51 ± 4 ***	97 ± 3	10 ± 1
Apicidin	183506-66-3 / LKT-A6132-M001	*Fusarium* sp.	53 ± 2 ***		55 ± 8 ***	63 ± 2 ***	25 ± 2 ***
Beauvericin	26048-05-5 / BV-B1246-1	*Beauveria bassiana*	30 ± 4 ***	*	50 ± 3 ***	115 ± 4 *	10 ± 1
Brefeldin A	20350-15-6 / LKT-B6816-M005	*Eupenicillium brefeldianum*	22 ± 1 ***	***	63 ± 4 ***	127 ± 5 ***	6 ± 1
Chaetocin	28097-03-2 / BV-2283-500	*Chaetomium* sp.	54 ± 3 ***		58 ± 5 ***	87 ± 2 *	15 ± 2
Citreoviridin	25425-12-1 / LKT-C3576-M001	*Penicillium citreoviride*	57 ± 1 ***		61 ± 4 ***	93 ± 3 *	11 ± 1
Citrinin	518-75-2 / BML-CM116-0005	*Penicillium citrinum*	72 ± 2 ***	*	48 ± 6 ***	85 ± 2 **	23 ± 1 ***
Cyclopiazonic acid	18172-33-3 / BML-CA415-0010	*Penicillium* sp.	65 ± 6 ***		53 ± 3 ***	104 ± 2	10 ± 1
Cyclosporin A	59865-13-3 / BML-A195-0100	*Fusarium solani*	51 ± 2 ***	*	81 ± 5 **	76 ± 1 ***	27 ± 3 ***
Cyclosporin B	63775-95-1 / LKT-C9615-M001	*Trichoderma polysporum*	33 ± 3 ***	*	94 ± 10	105 ± 4	8 ± 1
Cyclosporin C	59787-61-0 / LKT-C9612-M001	*Fusarium solani*	35 ± 4 ***	*	59 ± 2 ***	24 ± 3 ***	45 ± 2 ***
Cyclosporin D	63775-96-2 / BML-T109-0001	*Fusarium solani*	24 ± 1 ***	*	73 ± 11 ***	76 ± 2 ***	17 ± 2 **
Cyclosporin H	83602-39-5 / LKT-C9614-M001	*Tolypocladium inflatum*	35 ± 2 ***	*	48 ± 6 ***	104 ± 3	8 ± 1
Cytochalasin A	14110-64-6 / LKT-C9878-M001	*Drechslera dematoidea*	37 ± 2 ***	***	89 ± 4	75 ± 3 ***	25 ± 3 ***
Cytochalasin B	14930-96-2 / BML-T108-0005	*Drechslera dematoidea*	55 ± 3 ***	*	39 ± 4 ***	106 ± 2	9 ± 1
Cytochalasin C	22144-76-9 / LKT-C9880-M001	*Metarhizium anisopliae*	64 ± 1 ***		69 ± 5 ***	45 ± 3 ***	39 ± 1 ***
Cytochalasin D	22144-77-0 / LKT-C9881-M001	*Zygosporium mansonii*	75 ± 4 ***		75 ± 4 ***	93 ± 3 *	13 ± 1
Cytochalasin E	36011-19-5 / LKT-C9882-M001	*Aspergillus clavatus*	24 ± 3 ***	*	42 ± 3 ***	93 ± 3 *	19 ± 1 ***
Deoxynivalenol	51481-10-8 / ALX-630-115-M001	*Fusarium* sp.	34 ± 2 ***	*	67 ± 6 ***	64 ± 3 ***	24 ± 2 ***
Diacetoxyscripenol	2270-40-8 / LKT-D3200-M001	*Fusarium* sp.	46 ± 2 ***	*	100 ± 11	18 ± 1 ***	69 ± 2 ***
Fumagillin	23110-15-8 / LKT-C9882-M001	*Aspergillus fumigatus*	85 ± 2 ***	*	55 ± 6 ***	75 ± 3 ***	23 ± 4 ***
Fumigaclavine A	6879-59-0 / ALX-630-110-M001	*Aspergillus* sp.	65 ± 4 ***		75 ± 4 ***	49 ± 8 ***	34 ± 2 ***
Fumonisin B1	116355-83-0 / BML-SL220-0001	*Fusarium moniliforme*	40 ± 2 ***		49 ± 8 ***	93 ± 2	12 ± 2
Fumonisin B2	116355-84-1 / BML-SL219-0001	*Aspergillus niger*	13 ± 2 ***	**	59 ± 4 ***	106 ± 3	7 ± 1
Fusarenon X	23255-69-8 / LKT-F8272-M001	*Fusarium* sp.	64 ± 3 ***		67 ± 5 ***	94 ± 2	12 ± 2
Gliotoxin	67-99-2 / BML-PI129-0002	*Gladiocladium fimbriatum*	24 ± 2 ***	**	41 ± 2 ***	78 ± 4 ***	31 ± 1 ***
HC toxin	83209-65-8 / BML-GR320-0001	*Cochliobolus carbonum*	31 ± 2 ***	*	47 ±5 ***	66 ± 3 ***	23 ± 2 ***
HT-2 toxin	26934-87-2 / ALX-630-113-M001	*Fusarium tricinctum* (*)	86 ± 2 **	**	55 ± 4 ***	107 ± 4	8 ± 1
Moniliformin	31876-38-7 / LKT-M5853-M001	*Fusarium* sp.	60 ± 5 ***	*	86 ± 5 *	96 ± 3	11 ± 1
Moniliformin sodium salt	71376-34-6 / ALX-630-111-M001	*Fusarium moniliforme*	54 ± 2 ***	*	86 ± 6 *	54 ± 2 ***	35 ± 3 ***
Mycophenolic acid	24280-93-1 / BML-A249-0100	*Penicillium brevi-compactum*	39 ± 3 ***	*	62 ± 7 ***	99 ±2	18 ± 1 ***
Neosolaniol	36519-25-2 / LKT-N1858-M001	*Fusarium* sp.	34 ± 3 ***	*	60 ± 4 ***	56 ± 4 ***	35 ± 2 ***
Ochratoxin A	303-47-9 / ALX-630-089-M001	*Aspergillus ochraceus*	22 ± 1 ***	**	63 ± 4 ***	99 ± 3	13 ± 2
Ochratoxin B	4825-86-9	*Aspergillus* sp.	34 ± 2 ***	*	75 ± 8 ***	93 ± 3 *	16 ± 2 *
Patulin	149-29-1 / ALX-270-111-M001	*Penicillium expansum*	17 ± 3 ***	*	45 ± 7 ***	106 ±4	13 ± 2
Paxilline	57186-25-1 / BML-KC155-0005	*Penicillium paxilli*	24 ± 2 ***	**	73 ± 4 ***	55 ± 3 ***	32 ± 2 ***
Penitrem A	12627-35-9 / BML-KC157-0001	*Penicillium palitans*	15 ± 1 ***	**	43 ± 2 ***	66 ± 2 ***	24 ± 2 ***
Phomopsin A	12627-35-9 / ALX-350-417-M001	*Phomopsis leptostromiformis*	35 ± 2 ***	**	68 ± 4 ***	110 ± 6	9 ± 2
Roquefortine C	58735-64-1 / ALX-350-342-MC05	*Penicillium roqueforti*	79 ± 2 ***	**	56 ± 3 ***	76 ± 3 ***	26 ± 1 ***
Skyrin	602-06-2 / BV-2043-1	*Talaromyces* sp.	34 ± 2 ***	*	59 ± 4 ***	113 ± 4 *	6 ± 1
Stachybotrylactam	163391-76-2 / ALX-630-112-M005	*Stachybotrys* sp.	17 ± 1 ***	*	37 ± 6 ***	85 ± 2 **	18 ± 2 ***
Sterigmatocystin	10048-13-2 / ALX-630-116-M001	*Aspergillus versicolor*	24 ± 2 ***	***	63 ± 3 ***	54 ± 2 ***	32 ± 2 ***
Strobilurin B	65105-52-4 / ALX-380-144-M001	*Strobilurus* sp.	59 ± 2 ***		58 ± 13 ***	106 ± 4	8 ± 2
T2 tetraol	34114-99-3 / LKT-T0003-M001	*Fusarium* sp.	17 ± 2 ***	*	35 ± 3 ***	122 ± 3 **	7 ± 2
T2 toxin	21259-20-1 / ALX-630-101-M001	*Fusarium tricinctum*	57 ± 4 ***	*	84 ± 6 *	87 ± 3 *	24 ± 3 ***
T2 triol	34114-98-2 / LKT-T0004-M001	*Fusarium* sp.	75 ± 2 ***		84 ± 15 *	96 ± 2	11 ± 1
Tenuazonic acid	610-88-8 / ALX-350-317-MC05	*Alternaria* sp.	53 ± 2 ***	**	71 ± 2 ***	65 ± 4 ***	35 ± 3 ***
Territrem B	70407-20-4 / ALX-630-117-MC05	*Aspergillus terreus*	40 ± 2 ***	**	76 ± 4 ***	103 ± 2	8 ± 2
Verruculogen	12771-72-1 / LKT-V1870-M001	*Penicillium verruculosum*	16 ± 3 ***	**	140 ± 12 ***	94 ± 3	13 ± 1
Wortmannin	19545-26-7 / LKT-W5769-M001	*Penicillium wortmannii*	85 ± 3 ***	***	39 ± 2 ***	79 ± 2 ***	21 ± 2 ***
Zearalenone	17924-92-4 / ALX-630-105-M010	*Giberella zeae*	91 ± 3 *	**	40 ± 4 ***	94 ± 3	14 ± 2
α-Zearalanol	26538-44-3 / LKT-Z161022-M001	*Fusarium* sp.	87 ± 2 *	*	49 ± 7 ***	110 ± 4	9 ± 1

Tested mycotoxins were added to the cell cultures at final concentrations of 1 ng/ml (Sf-9 and Caco-2) and 1 μg/ml (THP-1). Proliferation tests were performed using Cell Counting Kit-8 (Abbkine) as described in Materials and methods. Cell mortality tests were performed on non-adherent THP-1 cells using propidium iodide and Arthur cell counter (NanoEnTek). Control C2—ethanol treated cells. Each control was performed in 20 independent replications. The effect of each mycotoxin on each cell line was tested in 3 independent replications. The results are presented as a percentage of control C2 findings, assumed to be 100%. All mycotoxins were purchased via the Axxora platform (http://www.axxora.com). (*) Semisynthetic, derived from T2 toxin from *Fusarium tricinctum*. Statistical significance:

* p < 0.05

** p < 0.001

*** p < 0.0001. Dunnett test was applied for mycotoxins-to-control comparisons while *t* test was used to compare the reactivity of Sf-9 cells with Caco-2 cells.

In the case of Caco-2 cells, no effect on cell proliferation was found after application of aflatoxin M2, alternariol (which exerted potent effect on Sf-9 cells), γ-ammanitin, cyclosporine B, cytochalasin A or diacetoxyscripenol. It is noteworthy that verruculogen, which inhibited the proliferation of Sf-9 cells to 16 ± 3%, had the opposite effect on Caco-2 (140 ± 12% of cell proliferation). The lowest values of Caco-2 cell proliferation were measured after the application of T2 tetraol (35 ± 3%), stachybotrylactam (37 ± 6%), cytochalasin B (39 ± 4%) or wortmannin (39 ± 2%). Application of these four mycotoxins to Sf-9 cells exerted either a similar effect, i.e. strong inhibition of cell proliferation (T2 tetraol, stachybotrylactam), or slightly decreased proliferation (cytochalasin B, wortmannin). The following mycotoxins strongly decreased the proliferation of Sf-9 cells (drop to 13–30%) but displayed a moderate or no effect on the proliferation of Caco-2 cells: 3-acetyldeoxynivalenol, alternariol, alternariol-9-methyl ether, brefeldin A, cyclosporines B and C, ochratoxin A, paxilline, sterigmatocystin, and verruculogen (which stimulated proliferation of Caco-2 cells; Table 1).

In the case of THP-1 cells, the tested mycotoxins had to be applied at 1000-times higher doses (1 μg/ml versus 1 ng/ml applied to Sf-9 and Caco-2 cell cultures) before significant effect on cell viability was observed. A significant decrease in THP-1 cell viability was observed only after the administration of the 0.5 and 1 μg/ml doses for four mycotoxins randomly selected from the 65 tested compounds: alternariol, deoxynivalenol, aflatoxin G2 and HC toxin (Fig 1, S1 Table).

**Fig 2:**
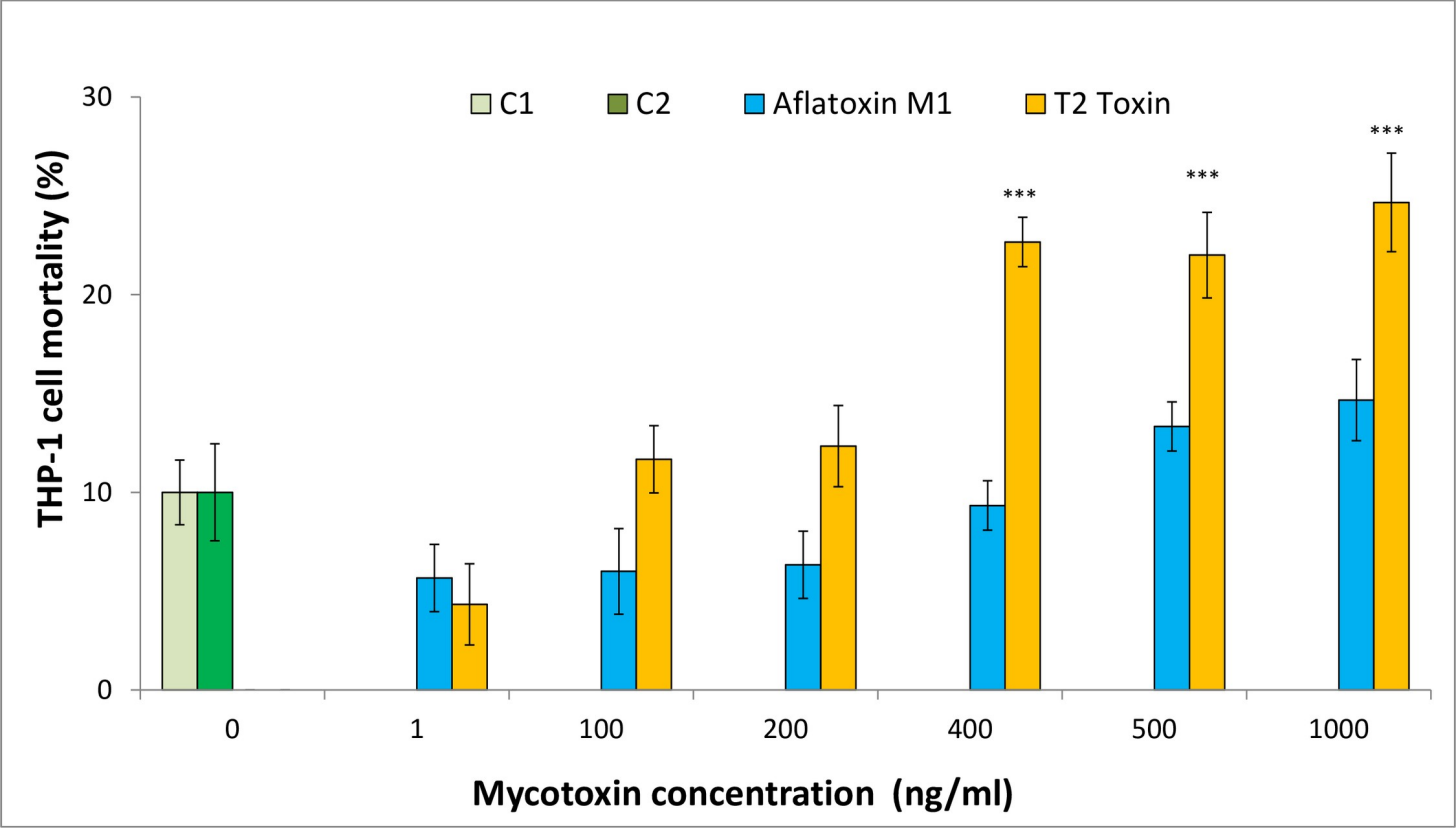
Dose-response effects of aflatoxin M1 and T2-toxin on the mortality of THP-1 cells *in vitro*. The statistical significance of the differences observed between treated cultures and controls at all concentrations of each tested mycotoxin were tested using Dunnett test. Each test was performed in 3 independent replications; controls: C1 (no treatment) and C2 (solvent treatment), each in 3 replications. Statistical significance: * p < 0.05, ** p <0.001, *** p <0.0001.

The enormous resistance of THP-1 cells to mycotoxins is evidenced by the fact that the increase in mortality after administration of T2-toxin was observed only at concentrations of 400 ng/ml and more, and the complete lack of cell mortality elevation after administration of aflatoxin M1, even at the concentration of 1 μg/ml (Fig 2, S2 Table).

No effect on THP-1 cell proliferation was observed for 22 mycotoxins administered at such a high concentration: aflatoxins B2 and M2, antibiotic PF 1052, cyclopiazonic acid, cyclosporine B and H, cytochalasin B, fumonisin B1 and B2, fusarenon X, HT-2 toxin, moniliformin, mycophenolic acid, ochratoxin A, patulin, phomopsin A, strobilurin B, T2 triol, territrem B, verruculogen, zearalenone and α-zearalanol (Table 1). THP-1 cell proliferation was found to be stimulated following administration of 3-acetyldeoxynivalenol, beauvericin, brefeldin A, skyrin, and T2 tetraol. Inhibition of THP-1 cell proliferation was only associated with aflatoxicol, cytochalasin C and diacetoxyscirpenol.

Elevated THP-1 cell mortality was induced by 33 mycotoxins (Table 1), with the highest mortality associated with diacetoxyscirpenol (69 ± 2%), cyclosporine C (45 ± 2%), and cytochalasin C (39 ± 1%).

### Mycotoxin-induced morphological changes in insect and mammalian cells

**Fig 3:**
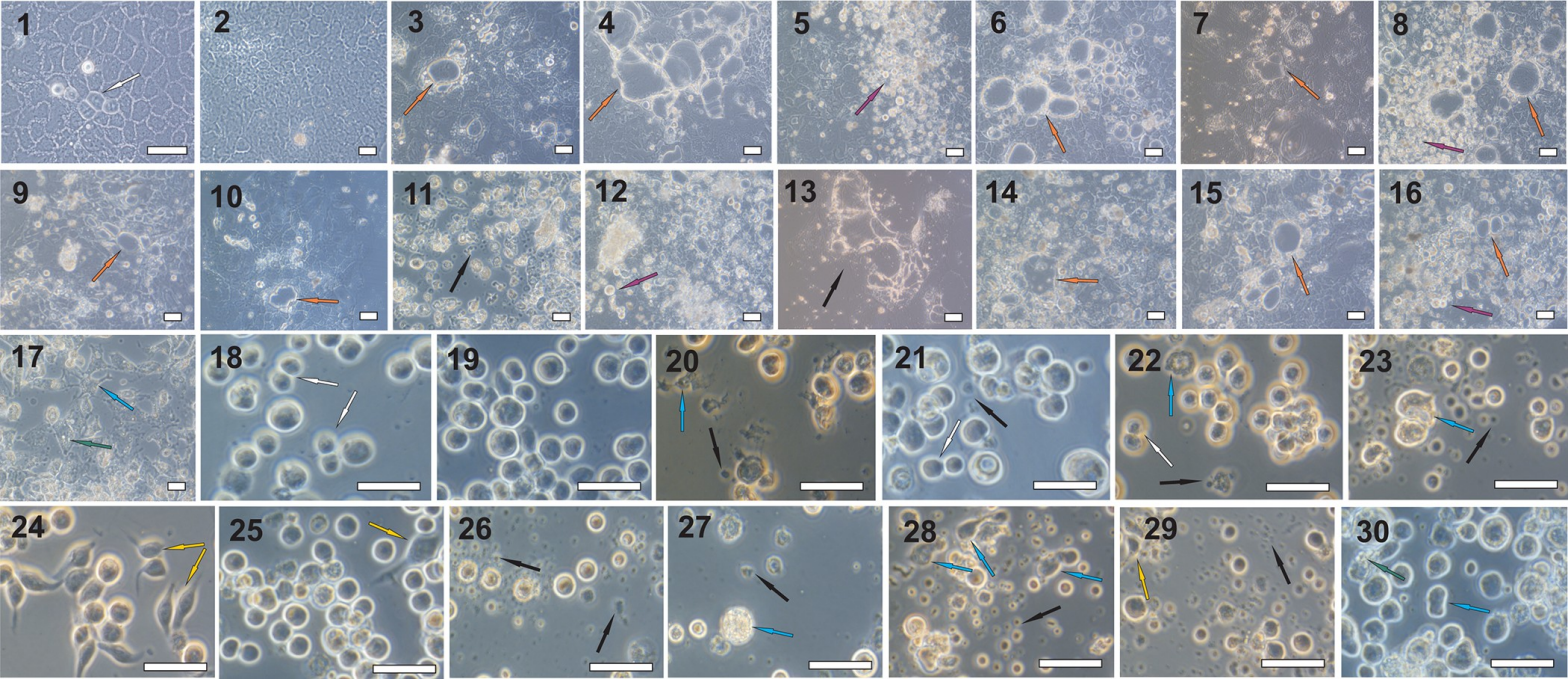
Examples of characteristic mycotoxin-induced morphological changes in Caco-2 and Sf-9 cells. (1) untreated Caco-2 control—C1, (2) Caco-2 control treated with solvent—C2, (3) Caco-2 treated aflatoxicol, (4) Caco-2 treated 3-acetyldeoxynivalenol, (5) Caco-2 treated aflatoxin B1, (6) Caco-2 treated aflatoxin G2, (7) Caco-2 treated α-amanitin, (8) Caco-2 treated antibiotic PF 1052, (9) Caco-2 treated cyclosporin H, (10) Caco-2 treated cytochalasin B, (11) Caco-2 treated gliotoxin, (12) Caco-2 treated HT-2 toxin, (13) Caco-2 treated roquefortine C, (14) Caco-2 treated stachybotrylactam, (15) Caco-2 treated T2 tetraol, (16) Caco-2 treated wortmannin, (17) Caco-2 treated zearalenone, (18) untreated Sf-9—C1, (19) Sf-9 treated with solvent—C2, (20) Sf-9 treated 3-acetyldeoxynivalenol, (21) Sf-9 treated aflatoxin B2, (22) Sf-9 treated alternariol, (23) Sf-9 treated alternariol-9-methyl ether, (24) Sf-9 treated fumonisin B2, (25) Sf-9 treated neosolaniol, (26) Sf-9 treated ochratoxin A, (27) Sf-9 treated patulin, (28) Sf-9 treated stachybotrylactam, (29) Sf-9 treated T2 tetraol, (30) Sf-9 treated verruculogen. White arrow—mitotic cells, yellow arrow—spindle cell, green arrow—vacuoles, blue arrow—malformed cell, orange arrow—monolayer surface protrusions, purple arrow—cells detached from the monolayer, black arrow—fragments of disintegrated cells. Scale bars: 50 μm.

Fig 3 shows examples of characteristic mycotoxin-induced morphological changes observed in Sf-9 and Caco-2 cells. The Sf-9 cultures were found to display disintegrating cells and varying degrees of cell fragmentation following treatment with all 65 tested mycotoxins. In addition, malformed cells were frequently seen, including swollen oval cells (patulin treatment), elongated cells with some kind of bubbles on the surface (stachybotrylactam treatment), swollen shapeless cells (alternariol-9-methyl ether treatment), cells with irregular shapes (stachybotrylactam and verruculogen treatments), cells with enlarged, shapeless nuclei (alternariol treatment), spread cells with irregular shaped protrusions (3-acetyldeoxynivalenol treatment), and spindle cells (fumonisin B2 and neosolaniol treatments). It should be noted that some examples of mitotic divisions of Sf-9 cells were observed in cultures containing disintegrated and distorted cells. Dividing cells were observed after administration of aflatoxin B2 and alternariol. Intensive cell vacuolization was seen after verruculogen administration.

Control Caco-2 cells showed a typical confluent monolayer appearance i.e. an adherent cell culture in which all cells were in contact with other cells. Mitotic Caco-2 cells were observed in untreated cells (C1) only. In Caco-2 cells, mycotoxin treatments resulted in changes in the shape of the cells, loss of cellular junctions and the loss of cell adherence to the growth surface. Detached and disintegrated cells were seen after administration of aflatoxin B1, antibiotic PF 1052, HT-2 toxin and wortmannin. Treatment of Caco-2 cells with roquefortine C resulted in complete disintegration of the monolayer and very rapid cell fragmentation. The addition of zearalenone induced monolayer disintegration and the appearance of highly-vacuolized cells which displayed a range of shapes, including characteristic starfish-like cells. The most frequently-observed change was the formation of monolayer surface protrusions: convex structures similar to blisters. Such structures, of various shapes and sizes, were observed after the administration of aflatoxicol, 3- acetyldeoxynivalenol, aflatoxin G2, α-amanitin, antibiotic PF 1052, cyclosporin H, cytochalasin B, stachybotrylactam, T2 tetraol, and wortmannin.

**Fig 4:**
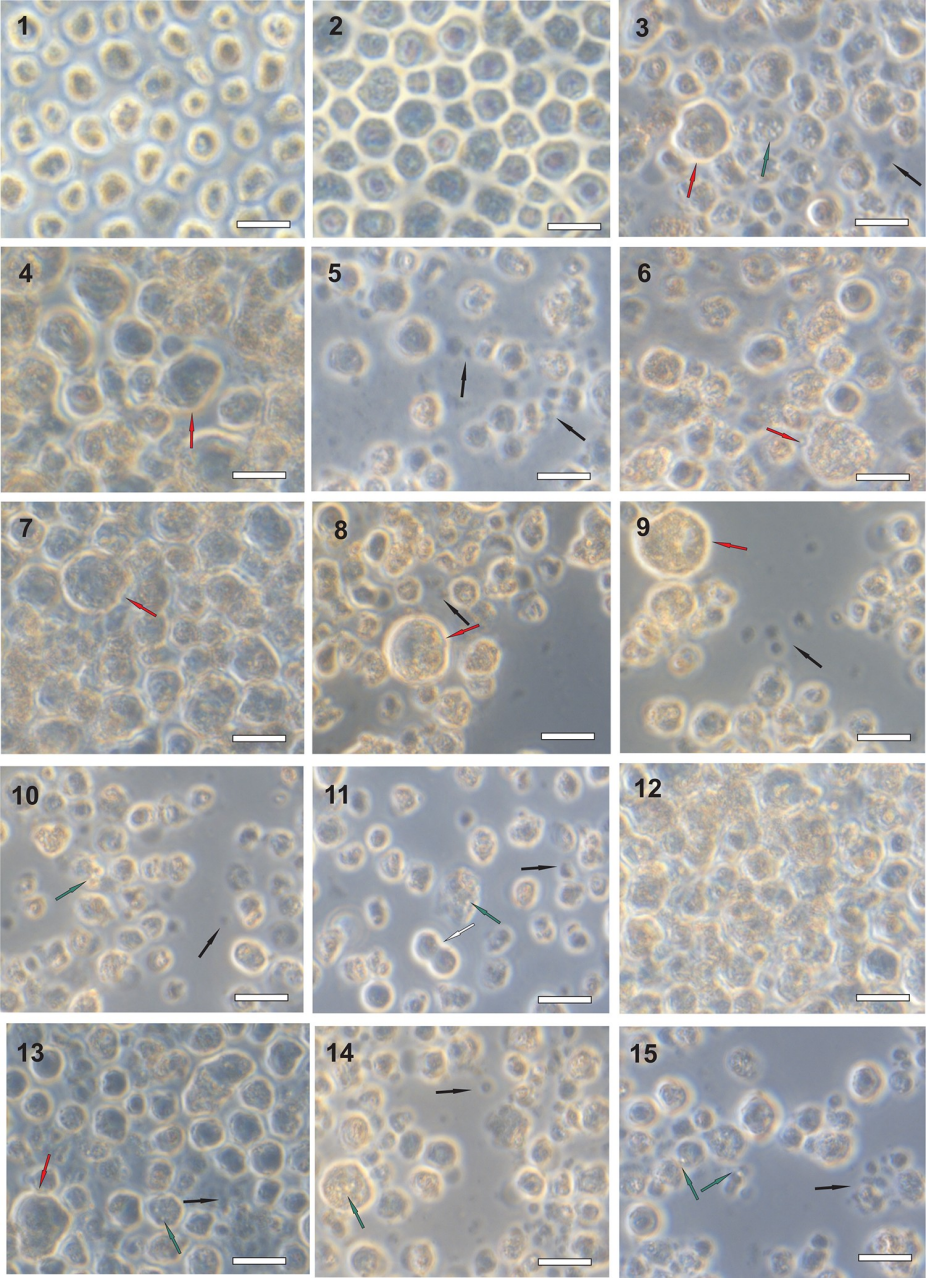
Examples of characteristic mycotoxin-induced morphological changes in THP-1 cells. (1) untreated THP-1 control—C1, (2) THP-1 control treated with solvent—C2, (3) aflatoxicol, (4) aflatoxin B2, (5) aflatoxin G2, (6) cyclosporin C, (7) cyclosporin H, (8) cytochalasin C, (9) diacetoxyscripenol, (10) fumigaclavine A, (11) moniliformin, (12) ochratoxin A, (13) patulin, (14) paxilline, (15) sterigmatocystin. White arrow—mitotic cells, red arrow—swelled and deformed cell, green arrow—vacuoles, black arrow—fragments of disintegrated cells. Scale bars: 50 μm.

THP-1 cells were found to be much more resistant to mycotoxins than Sf-9 and Caco-2 cells, as a 1000-fold greater dose was typically required to elicit a response. Typical mycotoxin-induced changes in THP-1 cell morphology are presented in Fig 4. Such swollen and deformed cells were seen after treatment with aflatoxicol, aflatoxin B2, cyclosporin C, cyclosporin H, cytochalasin C, diacetoxyscripenol, patulin and paxilline. Disintegrated and distorted cells were observed in cultures treated with aflatoxin G2, cytochalasin C, diacetoxyscripenol, fumigaclavine A, moniliformin, patulin, paxilline and sterigmatocystin. Vacuolized THP-1 cells appeared after the application of aflatoxicol, fumigaclavine A, moniliformin, patulin, paxilline and sterigmatocystin. Treatment with aflatoxin G2 resulted in the mass disintegration of cells, while the administration of ochratoxin A caused the cells to clump together. Moniliformin treatment induced vacuolization and cell disintegration, but cell divisions appeared to continue.

## Discussion

The damage caused to crops and stored food by harmful insects, as well as their transmission of pathogens causing serious diseases for humans, animals and plants require more effective control of pest populations. The insecticides currently used for this purpose are typically synthetic ones based on non-selective chemical compounds which accumulate in the environment and pose a serious threat to bio-diversity; growing pressure to minimize these deleterious effects has led to increased interest in the potential to harness the natural enemies of insects. Among various potential biocontrol agents, entomopathogenic fungi seem to be the most promising [18, 19].

The fungal pathogenesis of insects typically follows a series of events comprising conidial adhesion to the insect host cuticle, followed by penetration of the cuticle via fungal enzymes, the impairment of host immune responses, dissemination within the host and transmission from the host [19, 20]. Toxic metabolites released by the fungus into the hemocoel of the insect play an important role in this multi-step process; although the exact function of these fungal toxins is not always clear, it has been proposed that mycotoxins could be released directly to poison and kill the infected host or to suppress its immune system, thus facilitating the development of the mycosis [21]. Mycotoxins can also enter the interior of the insect body through the digestive tract, as fungi are desirable sources of food rich in proteins and sterols [22].

Numerous mycotoxins, with various chemical structures, show strong insecticidal activity against insect pests. Four decades ago, the insecticidal activities of aflatoxin B1, rubratoxin B, patulin and diacetoxyscirpenol have been examined in contact tests against *Drosophila melanogaster* [23], as has beauvericin against *Calliphora erythrocephala*, *Aedes aegypti*, *Lygus* spp., *Spodoptera frugiperda* and *Schizaphis graminum* [24]. Fungal ribotoxins, i.e. extracellular highly specific ribonucleases, were found to efficiently kill *Galleria mellonella* larvae and to display toxicity against two different insect cell lines: Sf-9 from *Spodoptera frugiperda* and Tni High Five from *Trichoplusia ni* [25]. In addition, tolypin, produced by two *Tolypocladium* species, was toxic to insects upon injection [26], leucinostins and efrapeptins, linear peptide toxins showing ATPase inhibitory activity, were active in foliar spray assays against *Leptinotarsa decemlineata* [27], and destruxins were found to display a strong lethal action against insects acting through a range of harmful processes on the cellular level [19]. Another study found a range of 22 mycotoxins to have a strong influence on the growth and mortality of *Helicoverpa zea* and *Spodoptera frugiperda*: aflatoxin B1, ochratoxin A, sterigmatocistin, citrinin, cyclopiazonic acid, penicillic acid, deoxynivalenol, diacetoxyscripenol, dihydrodeoxynivalenol, dihydroxycalonectrin, hydroxycalonectrin, moniliformion, sambucinol, T2 toxin, zearalenone, chaetoglobosin C, cytochalasin H, paspaline, paxilline, penitrem A, roseotoxin B, and verruculogen [22]. The action of aflatoxin B1 and T2 toxin against insects has been particularly widely studied; it has been found that while cockroaches are relatively resistant to aflatoxin B1, stored-product insects are relatively resistant to T2 toxin, and while *Drosophila melanogaster* is susceptible to aflatoxin B1, the larvae of *Bombyx mori*, *Heliotis zea* and *Spodoptera frugiperda* are sensitive to both aflatoxin B1 and T2 toxin [22]. Interestingly, many mycophagous species of *Drosophila* can tolerate the fungal poison α-amanitin in wild mushrooms and in artificial diet [28]. The activities of other lesser known insecticidal mycotoxins have been reviewed in other papers [29, 30]. Due to the tremendous variety of secondary metabolites produced by fungi, intensive studies concerning the insecticidal potential of mycotoxins are needed. As it is costly and time-consuming to use insects to test the insecticidal potential of mycotoxins, screening studies can be conducted more efficiently *in vitro* using insect cell lines [31].

Our data clearly show that all 65 tested mycotoxins significantly reduced the proliferation of insect Sf-9 cells *in vitro*. Sixteen of the mycotoxins used in the present study against the Sf-9 cells, including beauvericin, deoxynivalenol, diacetoxyscripenol, fusarenon X, fumonisin B1, gliotoxin, moniliformin, ochratoxin A and zearalenone (Table 1), have previously been tested by Fornelli and co-workers [31]. After converting our doses (1 ng/ml) to the micromolar concentrations used in the previous study, it was found that similar results were yielded only for beauvericin and gliotoxin; our Sf-9 cultures seem to be less sensitive to diacetoxyscripenol and fusarenon X than observed by Farnelli et al [31]. However, our cells were much more sensitive to deoxynivalenol, fumonisin B1, moniliformin, ochratoxin A and zearalenone. These discrepancies might result from the use of different tests to measure cell proliferation: although both tests are based on the activity of the mitochondrial succinate–tetrazolium reductase system, the two studies used different substrates (MTT versus WST-8).

The insect cell line Sf-9 is widely used to express recombinant proteins as the cultures are inexpensive and easy to scale up. However, care should be taken while growing Sf-9 cells, as they are extremely sensitive to fluctuations in temperature, cell density and agitation [32]. The application of fumonisin B2 to the Sf-9 cell cultures in the present study inhibited cell proliferation and resulted in the appearance of spindle cells (Table 1, Fig 2). Similar effects were reported by Zhang and co-workers, who report Sf-9 cell growth to be arrested at the G_2_/M phase, loss of adhesion, swelling and vacuole formation, depolarization of the cell membrane potential and hyperpolarization of the mitochondrial membrane potential following fumonisin B1 treatment [33]. The difference in susceptibility of the insect Sf-9 cells against the tested mycotoxins compared with that of the mammalian Caco-2 and THP-1 cells can be attributed to differences in cell lipid composition. Compared to mammalian cells, Sf-9 cells have reduced levels of sphingomyelin and elevated levels of phosphatidylethanolamine, and even when grown in mammalian serum, the plasma membranes of Sf-9 cells display very low cholesterol content and correspondingly low cholesterol to phospholipid ratio [34]. *In vitro* cultured insect cells have also demonstrated higher sensitivity to cholera toxin than tested mammalian cells [35].

Our research has shown that the THP-1 cells are extremely resistant to most tested mycototoxins. Furthermore, some mycotoxins, including 3-acetyldeoxynivalenol, beauvericin, brefeldin A, skyrin and T2 tetraol, stimulate the proliferation of THP-1 cells. The THP-1 cell line has been reported to be resistant to several drugs, and monocytic THP-1 human cell lines display different susceptibility to engineered nanoparticles compared to intestinal epithelial Caco-2 [36, 37]. Caco-2 has also been found to be more sensitive to cyclopiazonic acids than THP-1 cells [38], which is consistent with our observations (Table 1). However, more research is required to fully identify the reasons for the considerable difference observed between insect cells and the two types of mammalian cells to the same mycotoxins; despite progress in this area, the mechanisms underlying the disruptive influence of mycotoxins on the cellular machinery remain unraveled [39, 40].

Most studies focus on the major *Fusarium* mycotoxins which have been shown to cause a broad variety of toxic effects in animals. Trichothecenes, small amphipathic molecules that passively move across cell membranes, display multiple inhibitory effects on the primary metabolism of eukaryotic cells, including the inhibition of protein, DNA and RNA synthesis; in tissues with high cell turnover rates, such inhibition results in the alteration in cell proliferation [40, 41]. At the cellular level deoxynivalenol activates mitogen-activated protein kinase (MAPK) by a mechanism called the *ribotoxic stress response*, which drives both cytokine gene expression and apoptosis in macrophages [42]. Nivalenol and deoxynivalenol, which regularly co-occur in nature, share highly similar chemical structures and many toxicological properties, such as the inhibition of cell proliferation, induction of interleukin-8 secretion, and the involvement of MAPKs and nuclear factor κΒ in the signal transduction pathways associated with toxicities [40]. Zearalenone has been shown to be immunotoxic, hepatotoxic and nephrotoxic and an enhancer of lipid peroxidation; however, because of its structural similarity with the estrogen hormones, the major target of zearalenone is the mammalian reproductive system [40, 43]. Fumonisins, neurodegenerative mycotoxins, interfere with the biosynthesis of sphingolipids by inhibiting ceramide synthase, which impairs the metabolism of arachidonic acid and leads to degeneration of the sphingolipid-rich tissues [40]. Beauvericin, a cyclic hexadepsipeptide showing antimicrobial, antiviral, anti-tumor, cytotoxic, ionophoric, apoptotic and immunosuppressive activities, also demonstrates strong insecticidal activity against a broad spectrum of insects. Beauvericin increases the permeability of biological membranes by forming a complex with essential cations (Ca^2+^, Na^+^, K^+^) and/or cation-selective channels in lipid membranes, which affects cell homeostasis and the uncoupling of oxidative phosphorylation [40, 44]. The molecular mechanism of action of moniliformin is still obscure; however, its structural similarity to pyruvate suggests that moniliformin affects energy metabolism via the inhibition of mitochondrial pyruvate and α-ketoglutarate oxidation during the Krebs cycle [40]. The major effect of T2 toxin is inhibition of protein synthesis leading to disruption of DNA and RNA synthesis. The mechanisms of action of the T2 toxin and its metabolites against animals and humans are still not clearly understood [40].

Our comparison of the impact of individual mycotoxins on the three examined cell lines has allowed the selection of several promising candidates for further evaluation as potential insecticides. The mycotoxins cyclosporin B and D, cytochalasin E, gliotoxin, HC toxin, paxilline, penitrem A, stachybotrylactam and verruculogen significantly decreased Sf-9 cell proliferation while displaying rather mild effects on mammalian cells. The best candidate for mycotoxin-based insecticide appears to be cyclosporin B, which is known to be toxic to mosquito larvae [45]. In contrast, nothing is known of the insecticidal activity of cyclosporin D: a weak immunosuppressor, as well as a potent inhibitor of tumor-promoting phorbol ester *in vivo* and Ca^2+^/calmodulin dependent elongation factor 2 phosphorylation *in vitro* [46]. A similar lack of insecticidal data exists regarding cytochalasin E, an actin microfilament depolymerizing agent which inhibits angiogenesis and tumor growth [47], as well as for gliotoxin, which has a range of cytotoxic activities: blockage of membrane thiol groups, inhibition of the chymotrypsin-like activity of the 20S proteasome, induction of apoptosis in macrophages and thymocytes, and the activation of transcription factor NF-κB in response to a variety of stimuli in lymphocytes, and displays anti-inflammatory and anti-tumor activity *in vivo* [48], HC toxin, a potent, cell-permeable histone deacetylase inhibitor which displays antiprotozoal and antineoplastic activity and induces cell cycle arrest and apoptosis in tumor cells [49], and paxilline, penitrem, stachybotrylactam and verruculogen which all act as selective K+ channel blockers [50–53].

Alternariol, ochratoxin A, patulin, and sterigmatocystin cannot be considered as future myco-insecticides, as despite being mycotoxins displaying high cytotoxicity against Sf-9 cells with minor impact on Caco-2 and THP-1 cells, they are nevertheless known to display high toxicity and carcinogenicity in animals and humans [54–57]; the same applies to fumonisin, proposed by Zand and co-workers as bio-insecticide [33]. In addition, brefeldin and T2 tetraol show little potential for the control of insect pests as they have been found to stimulate the proliferation of human monocytic *leukemia* THP-1 cells (Table 1). The next step, therefore, in the identification of possible insecticidal candidates would be to perform a series of *in vivo* tests investigating the action of cyclosporins B and D, cytochalasin E, gliotoxin, HC toxin, paxilline, penitrem A, stachybotrylactam, and verruculogen against a range of insect pests.

It is worth mentioning the limitations of research carried out on cell lines. The observed differences between the insect cell line and mammalian cell lines cannot be interpreted as true differences between insects and mammals and the characteristics of the cell lines might be not representative of those of the organism they originate from. Furthermore, while the genomes of wild insect populations are typically highly heterogeneous, the Sf-9 cell line *derived from* ovarian tissues of the fall army worm might be genetically homogeneus as it is in the case of Hi5 cells originating from *Trichoplusia ni* ovaries [58]. A comparative genomic analysis of *T*. *ni* with *Bombyx mori* revealed high levels of genome synteny; however, genome synteny analysis of *T*. *ni* and Hi5 (a *T*. *ni-*derived cell line) indicated the presence of extensive genome rearrangements in the cell line and provided the first genomic evidence of a high degree of instability in chromosomes in lepidopteran cell lines [59]. For this reason, studies carried out only on cell lines may not reflect the true insecticidal activity of the tested mycotoxins, as the findings, or lack of such, observed on cell lines might not reflect effects observed on whole organisms. This difference could be due to a number of possible reasons such as a lack of mycotoxin key receptors expressed on the surface of the cultured cells, which are present in differentiated cells, or that mycotoxins which can easily penetrate the cell membrane *in vitro* are not able to bypass the cuticle/peritrophic membrane, or that mycotoxins may not be processed by host enzymes *in vitro* or *in vivo*. Deleterious side effects, such as carcinogenic effects may also interfere with the response of the whole organism. Further experiments are necessary to analyze all these parameters. Using of another insect cell line and hemocytes (insect immunocompetent cells) will explain if the insecticidal effect is insect cell line and/or cell type specific.

## Supporting information

S1 Table Proliferation of THP-1 cells after treatment with various concentrations of four randomly-selected mycotoxins—raw data. (XLSX)

S2 Table Mortality of THP-1 cells after treatment with various concentrations of two randomly-selected mycotoxins. (XLSX)

S3 Table Proliferation of Caco-2 and Sf-9 cells treated with 65 mycotoxins—raw data. (XLSX)
